# Diagnostic Value of High-Resolution Computed Tomography Scan in COVID-19: Do We Need to Think Outside the Box?

**DOI:** 10.7759/cureus.15849

**Published:** 2021-06-23

**Authors:** Muhammad Sheharyar Khan, Muhammad Bilawal Abbas Janjua, Ali Murad Jamal, Shehrbano Qaiser, Aamna Attiq, Arsalan Raza, Mustafa Tauseef Razzaq, Assadullah A Bhatti, Nitasha Afzal, Aiman Zahra

**Affiliations:** 1 Internal Medicine, Rawalpindi Medical University, Rawalpindi, PAK; 2 Internal Medicine, Foundation University Medical College, Islamabad, PAK; 3 Internal Medicine, Rahbar Medical and Dental College, Lahore, PAK; 4 Internal Medicine, Rashid Latif Medical College, Lahore, PAK; 5 Radiology, University College of Medicine and Dentistry, Lahore, PAK; 6 Radiology, Sargoda Medical College, Sargoda, PAK

**Keywords:** coronavirus disease 2019 (covid-19), real-time polymerase chain reaction (rt-pcr), high-resolution ct scan, diagnostic value, computed tomography severity score (ctss) of chest

## Abstract

Background and objective

The ambiguous nature and high infectivity of the coronavirus disease 2019 (COVID-19) have caused soaring morbidity and mortality worldwide. Real-time polymerase chain reaction (RT-PCR) is preferred for detecting COVID-19. However, its poor sensitivity and the emerging use of high-resolution CT (HRCT) scan for disease severity make the use of RT-PCR quite obsolete. In light of this, our study aimed to explore the beneficial role of HRCT and compare the HRCT findings across various patient demographics and parameters.

Methods

This cross-sectional study included 100 patients with clinical suspicion of COVID-19. All patients underwent a chest HRCT scan preceded by RT-PCR testing. We used the CT severity score (CTSS) of the chest to calculate disease severity. Demographical data and results of radiological findings were tabulated and compared across RT-PCR positivity, age, and gender. Independent samples t-test and chi-square test were used to analyze the data.

Results

Glass ground opacity was the most prevalent finding in 99% of the patients, followed by lymph node involvement, consolidation, and crazy-paving pattern. Pleural effusion was observed in only 10% of the patients while pericardial effusion and hiatal hernia were present in 5%. In RT-PCR-positive patients, the posterior basal segment of the lower lobe of the right and left lungs were found to be dominantly involved; however, the upper and middle lobes of the right lung were more commonly involved than the left lung. The mean CTSS was significantly higher in patients aged above 50 years (p<0.001). The mean CTSS of RT-PCR-negative patients was higher than that of RT-PCR-positive patients (15.18 vs. 14.31, p=0.537).

Conclusion

RT-PCR has a limited role in the diagnosis of COVID-19. The HRCT scan can detect typical COVID-19 findings even in patients with negative RT-PCR results. Moreover, the use of HRCT scan in determining the disease severity and extent of lung damage can lead to a better assessment of critically ill patients.

## Introduction

The coronavirus disease 2019 (COVID-19) pandemic has severely paralyzed healthcare systems worldwide [1]. The highly transmissible virus belongs to a family of respiratory viruses and was named severe acute respiratory syndrome coronavirus 2 (SARS-CoV-2) [2]. COVID-19 can present as an asymptomatic disease, symptomatic illness (cough, altered taste, anosmia, and breathlessness), or manifest as various complications including acute lung injury, atypical pneumonia, cardiac injury, acute renal injury, and functional impairment [3]. All these complications ultimately translate into an increased risk of morbidity and mortality.

For diagnosing COVID-19 in suspected patients, a myriad of laboratory and radiological diagnostic modalities are available. These modalities include real-time polymerase chain reaction (RT-PCR), serological testing through enzyme link immunosorbent assay (ELISA), chest radiography, and high-resolution CT (HRCT) scan [4]. Currently, WHO has recommended the use of RT-PCR over serological testing for detecting SARS-CoV-2 [5]. However, this test has certain limitations that include cumbersome laboratory workup, time-consuming tests, high expenses, shortage of kits, and a low sensitivity ranging between 60-71% [5,6]. Chest HRCT scan has been proven to have a central role in detecting COVID-19 in patients with negative RT-PCR; it has also created a benchmark in detecting the disease severity and is significant for monitoring the treatment efficacy and disease prognosis. Chest HRCT scan can detect various features including consolidation, ground-glass opacities, crazy-paving patterns, lobar and segmental involvement of lungs, involvement of mediastinal lymph node, and extra-pulmonary manifestations such as pleural or pericardial effusions [5,7].

The CT severity score (CTSS) of the chest is used for assessing the disease severity and the extent of lung damage [8]. It includes radiological observation of the 20 regions of both lungs and notices maximally involved segments. The chest CTSS is defined as the sum of scores of each lung segmental region, ranging from 0-40 according to the severity [5,8]. Based on these HRCT scan findings and chest CTSS, the disease severity in suspected COVID-19 patients is graded from very low (mild disease) to very high (critical illness) [8].

In this study, we aim to explore and investigate the role of HRCT scan in COVID-19 while examining the prevalence of various pulmonary and extrapulmonary findings. We further aim to compare these findings among patients with positive and negative RT-PCR results. Lastly, we aim to assess the differences in mean CTSS across ages, genders, and RT-PCR positivity. The findings of our study would lead to the implementation of measures to ensure favorable outcomes in COVID-19 patients in terms of disease identification, stratification, treatment, prognosis, and assessment for severity.

## Materials and methods

This was a retrospective cross-sectional study conducted on 100 patients with clinical suspicion of COVID-19. The study was conducted at the Department of Radiology, Citi Laboratories in Rawalpindi, Pakistan from February to April 2021. The inclusion criteria were as follows: suspected COVID-19 patients with typical symptoms (cough, shortness of breath, documented fever, and body aches), oxygen saturation below 94%, and COVID-19 suspicion based on initial chest X-ray findings (peripheral pulmonary infiltrates and consolidation). The exclusion criteria consisted of patients having non-viral pneumonia, tuberculosis, asthma, chronic obstructive pulmonary disease, and pulmonary edema. These patients were excluded based on systemic examination and patient history. Data was collected using a self-designed proforma that included demographic details, RT-PCR results, and chest HRCT scan findings. All the patients underwent HRCT scans according to the standard protocol, and chest CTSS was calculated from HRCT scan findings.

The HRCT scan was obtained from a multidetector Toshiba Aquilion CT scanner (Canon Medical Systems Corporation, Otawara, Japan) with a tube voltage of 120 kbp and a current of 350 mA. The supine position was used to obtain the chest HRCT scan. All lung fields were completely assessed from apices to bases and a consultant radiologist (with experience of 10 years in the field) reported all the findings. Thereafter, chest CTSS was calculated from the HRCT scan findings, and findings in each lung segment were reported. Categorical variables were expressed as frequency and percentages, while continuous variables were described using mean ± standard deviations. Independent samples t-test and chi-square test were used to analyze the data. A p-value of below 0.05 was considered statistically significant. Data were entered and analyzed using SPSS Statistics version 23.0 (IBM, Armonk, NY).

## Results

Our cohort had a total of 100 patients; the mean age of the patients was 54.0 ± 14.0 years (range: 24-83 years). Of these 100 patients, 55 (55%) patients were male and 45 (45%) were females. In patients with proven RT-PCR positivity, there was a significant association with consolidation (88.5% vs. 63.6%, p=0.006) and lobe involvement (p=0.001). Cardiomegaly was more common with patients aged more than 50 years (51.6% vs. 21.1%, p=0.002). The comparison of HRCT findings across RT-PCR positivity is shown in Table 1.

**Table 1:** Comparison of HRCT scan findings between RT-PCR-positive and RT-PCR-negative patients RT-PCR: real-time polymerase chain reaction; HRCT: high-resolution computed tomography

Feature		RT-PCR-positive patients (n=78), n (%)	RT-PCR-negative patients (n=22), n (%)	Total (n=100), n (%)
Dominant lobe involvement	Right	47 (60.3%)	3 (13.6%)	50 (50.0%)
	Left	14 (17.9%)	0 (0.0%)	14 (14.0%)
	Equal	17 (21.8%)	19 (86.4%)	36 (36.0%)
Segment involvement	Upper	5 (6.4%)	3 (13.6%)	8 (8.0%)
	Middle	0 (0.0%)	0 (0.0%)	0 (0.0%)
	Lower	73 (93.6%)	19 (86.4%)	92 (92.0%)
Ground-glass appearance	Yes	77 (98.7%)	22 (100%)	99 (99.0%)
	No	1 (1.3%)	0 (0.0%)	1 (1.0%)
Consolidation	Yes	69 (88.5%)	14 (63.6%)	83 (83.0%)
	No	9 (11.5%)	8 (36.4%)	17 (17.0%)
Crazy-paving pattern	Yes	59 (75.6%)	15 (68.2%)	74 (74.0%)
	No	19 (24.4%)	7 (31.8%)	26 (26.0%)
Involvement of lymph nodes	Yes	76 (97.4%)	21 (95.5%)	97 (97.0%)
	No	1 (2.6%)	2 (4.5%)	3 (3.0%)
Pleural effusion	Yes	6 (7.7%)	4 (18.2%)	10 (10.0%)
	No	74 (92.3%)	18 (81.8%)	90 (90.0%)
Cardiomegaly	Yes	32 (41.0%)	8 (36.4%)	40 (40.0%)
	No	46 (59.0%)	14 (63.6%)	60 (60.0%)
Hiatal hernia	Yes	5 (6.4%)	0 (0.0%)	5 (5.0%)
	No	73 (93.6%)	22 (100.0%)	95 (95.0%)
Pericardial effusion	Yes	3 (3.8%)	2 (9.1%)	5 (5.0%)
	No	75 (96.2%)	20 (89.9%)	95 (95.0%)

The mean chest CTSS among the patients was 14.5 ± 5.83. The score showed a difference when stratified according to age above 50 years, gender, and RT-PCR positivity, as shown in Table 2.

**Table 2:** Comparison of chest CTSS across patient parameters *Independent samples t-test CTSS: computed tomography severity score; RT-PCR: real-time polymerase chain reaction; SD: standard deviation

Parameter		Mean chest CTSS ± SD	P-value*
Gender	Male	14.11 ± 5.5	0.462
	Female	14.98 ± 6.1	
RT-PCR positivity	Yes	14.31 ± 5.8	0.537
	No	15.18 ± 6.2	
Age above 50 years	Yes	16.21 ± 5.1	<0.001
	No	11.71 ± 4.8	

The posterior basal segments of the lower lungs showed the maximum level of involvement in RT-PCR-positive patients (n=78). This is illustrated in Table 3.

**Table 3:** A breakdown of lung involvement among 78 RT-PCR positive patients RT-PCR: real-time polymerase chain reaction

Lung lobe	Segment	Right, n (%)	Left, n (%)
Upper	Apical	53 (67.9%)	36 (46.2%)
	Anterior	46 (59.7%)	38 (48.7%)
	Posterior	67 (85.9%)	52 (66.7%)
	Lingula-superior	0 (0.0%)	53 (67.9%)
	Lingula-inferior	0 (0.0%)	65 (83.3%)
Middle	Lateral	57 (73.1%)	0 (0.0%)
	Medial	54 (69.2%)	0 (0.0%)
Lower	Superior	51 (65.4%)	50 (64.1%)
	Medial basal	57 (73.1%)	51 (65.4%)
	Anterior basal	48 (61.5%)	50 (64.1%)
	Lateral basal	57 (73.1%)	59 (75.6%)
	Posterior basal	70 (89.7%)	70 (89.7%)

## Discussion

Our study showed that the most common features detected even in RT-PCR-negative patients were ground-glass appearance and consolidation. A study from Pakistan has reported similar findings, with ground-glass appearance and consolidation among 88.5% and 52.8% of the patients respectively [5]. Similarly, a study conducted in Italy showed ground-glass appearance with or without consolidation in nearly 97% of the patients [9]. In our study, the crazy-paving pattern on HRCT scan was observed in 74% of the patients; however, other studies have reported its prevalence to be between 12.5-36% [10,11]. In our study, pleural and pericardial effusion were observed in 10% and 5% of the patients respectively. Other studies in the literature have also reported a similar prevalence: between 4.8-8.4% [11,12]. The literature substantiates the involvement of the right lower lobe more than the left lung [5,11]. Our study showed that the posterior basal segments of the lower lobes of both lungs were predominantly involved followed by posterior segments of the upper lobe of the right lungs. These findings are in line with another study that showed that lateral basal and medial basal segments of the right lung were predominantly involved in moderate-to-critical COVID-19 patients [13].

Our study has shown that the chest HRCT scan can detect typical findings of COVID-19 even in patients with negative RT-PCR results. Non-contrast HRCT scan is now a preferred diagnostic modality and plays a more crucial role in the diagnosis of clinically suspected COVID-19 patients [14]. Similarly, many studies have established that HRCT scan has a higher sensitivity of up to 85% and specificity of up to 88% as compared to RT-PCR for diagnosing COVID-19 [8,14,15]. In China, the National Health Commission authorities have also recommended the use of HRCT scan over RT-PCT for COVID-19 diagnosis due to its high accuracy [16]. Unfortunately, developing countries like Pakistan have not yet caught up and health professionals here mostly rely on RT-PCR positivity due to a lack of equipment and qualified radiologists. Raising awareness among healthcare professionals and encouraging the use of RT-PCR results along with chest HRCT scan results might offer a better solution to the problem.

Patients suffering from COVID-19 present with a vast array of symptoms ranging from asymptomatic disease to life-threatening cytokine storm [7]. The cytokine storm damages the pulmonary system, which can be life-threatening, especially in patients with multiple comorbidities and advanced age [17]. The results of our study are consistent with this finding as older patients had significantly higher chest CTSS than younger patients. In our study, it was observed that patients with positive RT-PCR had a lower mean chest CTSS as compared to patients with negative RT-PCR results. A study from Egypt has also shown similar results, by reporting a non-significant difference across positive and negative RT-PCR results [18]. Another study from Pakistan on chest HRCT scan findings in patients with RT-PCR-negative results has reported that 79.2% of patients had ground-glass opacities [19]. In comparison, our study reported ground-glass appearance in 98.7% of RT-PCR-positive COVID-19 patients and in 100% of RT-PCR-negative COVID-19 patients.

The major limitation of our study was its retrospective cross-sectional design based on a convenience sampling technique. We recommend that further cohort studies be conducted to delve deeper into the diagnostic role of chest HRCT in COVID-19 so that its superiority over RT-PCR can be conclusively established. There is an urgent need for the implementation of guidelines so that the diagnostic accuracy of COVID-19 can be established in a better way.

## Conclusions

Patients with RT-PCR-negative results may present with typical COVID-19 features on HRCT scan evaluation. Chest HRCT scan can delineate several pulmonary and extrapulmonary findings that aid in determining the disease severity. Upon HRCT scan evaluation, posterior basal and lateral basal segments of the lower lobe of the right lung were found to be dominantly involved. The comparative findings on HRCT scan and CTSS between positive and negative RT-PCR patients demonstrated non-significant differences. Hence, the use of a chest HRCT scan along with clinical correlations could offer a solution for low RT-PCR sensitivity in the diagnosis of COVID-19 and its severity.

## References

[1] Almas T, Ehtesham M, Khan AW (2021). Safety and efficacy of low-dose corticosteroids in patients with non-severe coronavirus disease 2019: a retrospective cohort study. Cureus.

[2] Hashim L, Khan HR, Ullah I (2020). Physician preparedness in response to the coronavirus disease 2019 pandemic: a cross-sectional study from a developing country. Cureus.

[3] Nicholson AG, Osborn M, Devaraj A, Wells AU (2020). COVID-19 related lung pathology: old patterns in new clothing?. Histopathology.

[4] Sun Z, Zhang N, Li Y, Xu X (2020). A systematic review of chest imaging findings in COVID-19. Quant Imaging Med Surg.

[5] Khaliq M, Raja R, Khan N, Hanif H (2020). An analysis of high-resolution computed tomography chest manifestations of COVID-19 patients in Pakistan. Cureus.

[6] Dai H, Zhang X, Xia J (2020). High-resolution chest CT features and clinical characteristics of patients infected with COVID-19 in Jiangsu, China. Int J Infect Dis.

[7] Guan CS, Lv ZB, Yan S (2020). Imaging features of coronavirus disease 2019 (COVID-19): evaluation on thin-section CT. Acad Radiol.

[8] Francone M, Iafrate F, Masci GM (2020). Chest CT score in COVID-19 patients: correlation with disease severity and short-term prognosis. Eur Radiol.

[9] Grassi R, Fusco R, Belfiore MP (2020). Coronavirus disease 2019 (COVID-19) in Italy: features on chest computed tomography using a structured report system. Sci Rep.

[10] Awulachew E, Diriba K, Anja A, Getu E, Belayneh F (2020). Computed tomography (CT) imaging features of patients with COVID-19: systematic review and meta-analysis. Radiol Res Pract.

[11] Li K, Wu J, Wu F, Guo D, Chen L, Fang Z, Li C (2020). The clinical and chest CT features associated with severe and critical COVID-19 pneumonia. Invest Radiol.

[12] Bao C, Liu X, Zhang H, Li Y, Liu J (2020). Coronavirus disease 2019 (COVID-19) CT findings: a systematic review and meta-analysis. J Am Coll Radiol.

[13] Zayed NE, Bessar MA, Lutfy S (2021). CO-RADS versus CT-SS scores in predicting severe COVID-19 patients: retrospective comparative study. Egypt J Bronchol.

[14] Zhou S, Wang Y, Zhu T, Xia L (2020). CT features of coronavirus disease 2019 (COVID-19) pneumonia in 62 patients in Wuhan, China. AJR Am J Roentgenol.

[15] Majidi H, Bani-Mostafavi ES, Mardanshahi Z, Godazandeh F, Ghasemian R, Heydari K, Alizadeh-Navaei R (2020). High-resolution computed tomography finding in 552 patients with symptomatic COVID-19: first report from north of Iran. Emerg Radiol.

[16] Bernheim A, Mei X, Huang M (2020). Chest CT findings in coronavirus disease-19 (COVID-19): relationship to duration of infection. Radiology.

[17] Chen N, Zhou M, Dong X (2020). Epidemiological and clinical characteristics of 99 cases of 2019 novel coronavirus pneumonia in Wuhan, China: a descriptive study. Lancet.

[18] Korkmaz I, Dikmen N, Keleş FO, Bal T (2021). Chest CT in COVID-19 pneumonia: correlations of imaging findings in clinically suspected but repeatedly RT-PCR test-negative patients. Egypt J Radiol Nucl Med.

[19] Yusuf S, Ahmad H, Zeb R, Zeb U, Zeb AA (2021). High-resolution CT chest findings in suspected COVID-19 pneumonia patients with negative real-time polymerase chain reaction assay. Cureus.

